# Partial-Body Cryostimulation Increases Resting Energy Expenditure in Lean and Obese Women

**DOI:** 10.3390/ijerph18084127

**Published:** 2021-04-14

**Authors:** Massimo De Nardi, Ambra Bisio, Lucio Della Guardia, Carlo Facheris, Emanuela Faelli, Antonio La Torre, Livio Luzi, Piero Ruggeri, Roberto Codella

**Affiliations:** 1 Krioplanet Ltd., Treviglio, 24047 Bergamo, Italy; info@krioplanet.it (M.D.N.); carlo.facheris91@gmail.com (C.F.); 2 Department of Experimental Medicine, Università Degli Studi di Genova, 16132 Genoa, Italy; ambra.bisio@unige.it (A.B.); emanuela.faelli@unige.it (E.F.); ruggeri@unige.it (P.R.); 3 Centro Polifunzionale di Scienze Motorie, Università Degli Studi di Genova, 16132 Genoa, Italy; 4 Department of Biomedical Sciences for Health, Università Degli Studi di Milano, 20133 Milano, Italy; lucio.dellaguardia@unimi.it (L.D.G.); antonio.latorre@unimi.it (A.L.T.); livio.luzi@unimi.it (L.L.); 5 IRCCS Istituto Ortopedico Galeazzi, 20161 Milan, Italy; 6 Department of Endocrinology, Nutrition and Metabolic Diseases, IRCCS MultiMedica, 20138 Milano, Italy

**Keywords:** cold therapy, white adipose tissue, metabolism, thermogenesis, weight loss, adipocytes, indirect calorimetry, skeletal muscle, obesity treatment, brown adipose tissue

## Abstract

Cryostimulation is currently seen as a potential adjuvant strategy to tackle obesity and dysmetabolism by triggering cold-induced thermogenesis. Although suggestive, the underlying mechanisms are still poorly elucidated. We tested whether single or repeated applications of partial-body cryostimulation (PBC) could influence resting energy expenditure (REE) in exposed individuals. Fifteen middle-aged obese and sixteen control lean women (body mass index 31 ± 1.6 kg/m^2^ and 22 ± 1.7 kg/m^2^) underwent a daily PBC (−130 °C × 150 s) for five consecutive days. Resting energy metabolism (REE) was assessed by indirect calorimetry pre- and post-PBC on day 1 and day 5. As concerns REE, the linear mixed model revealed that REE changes were explained by session and time (F_1,29_ = 5.58; *p* = 0.02; ƞ_p_^2^ = 0.16) independent of the group (F_1,29_ = 2.9; *p* = 0.09; ƞ_p_^2^ = 0.09). REE pre-PBC increased from day 1 to day 5 either in leans (by 8.2%, from 1538 ± 111 to 1665 ± 106 kcal/day) or in obese women (by 5.5%, from 1610 ± 110 to 1698 ± 142 vs kcal/day). Respiratory quotient was significantly affected by the time (F_1,29_ = 51.61; *p* < 0.000001, ƞ_p_^2^ = 0.64), as it increased from pre- to post-PBC, suggesting a shift in substrate oxidation. According to these preliminary data, cold-induced thermogenesis could be explored as a strategy to elevate REE in obese subjects. Longitudinal studies could test whether chronic PBC effects may entail favorable metabolic adaptations.

## 1. Introduction

Obesity constitutes a global health burden [1] and is defined as the overexpansion of white adipose tissue (WAT) leading to ponderal excess (BMI ≥ 30 kg/m^2^). It is commonly featured by a variable degree of low-grade inflammation and immune cells infiltration in WAT [2]. Beyond storing excess energy in the form of triglycerides, WAT functions as an endocrine organ, ensuring metabolic balance. Adipocytes are plastic cells [3] capable of adapting their biological profile according to environmental changes, in the effort to ensure energy homeostasis. When exposed to energy excess, adipocytes undergo morphological and biochemical changes, which promote cell dysfunction and the development of local and systemic inflammatory response [2].

In contrast to WAT, brown adipose tissue (BAT) catabolizes glucose and lipids for thermogenic purposes [4]. Active BAT deposits have been found in the supraclavicular and neck regions in humans [5]. Additionally, paravertebral, mediastinal, para-aortic and suprarenal area are interested by functional BAT deposits in adults [5]. Due to high plasticity, white adipocytes are capable to convert to brown-like adipocytes (and vice versa) in response to various stimuli like cold [4,5]. Thus, the redirection of white adipocytes or white precursors towards brown phenotype could contribute to control obesity [4,5].

Resting energy expenditure (REE) typically accounts for 60% to 75% of total daily energy expenditure and represents the amount of energy required to maintain systemic homeostasis [6]. Individuals with low REE are demonstrated to have an increased risk of developing obesity and related disorders [7,8,9]. Conversely, the increase of REE could represent a potential resource for weight-loss in obese subjects [10,11].

The increase in energy expenditure upon cold exposure has long been known [12]. Cold exposure increases REE by activating shivering (ST) and the non-shivering thermogenesis (NST) in a number of tissues including WAT, BAT and skeletal muscle. Cold exposure is believed to increase lipid catabolism and adipose tissue-related thermogenesis [10,12], although the mechanisms remain unclear. In humans, BAT increases its metabolic rate and cell number to maintain the body temperature in response to cold. The increased oxidative metabolism in BAT directly contributes to augmented energy expenditure upon cold exposure [10].

Partial body cryostimulation (PBC) consists in brief (60–180 s) exposure to a steam of liquid nitrogen (between −130 °C and −170 °C) of the whole body, except for the head and neck [13]. The effect of PBC on obese individuals still represents a poorly explored field of research. Growing attention is being given to this technique as suitable strategy to promote weight loss, ameliorate adipose tissue function and whole-body metabolic homeostasis [14,15] in obese individuals. In fact, PBC has been previously used to reduce pro-inflammatory response, relieve pain and enhance muscles’ post-exercise recovery; however, its use as an alternative strategy to trigger cold-induced thermogenesis remains unexplored [16].

The aim of present investigation is to characterize the REE response in lean and obese subjects undergoing PBC, as to providing the mechanistic background for further investigations on the topic.

## 2. Materials and Methods

### 2.1. Subjects and Design

Normal weight and obese middle-aged women were recruited for this study. Based on the a priori sample-sized determination (software G*Power 3.1.9.2, Universität Düsseldorf, Germany), with a statistical power of 0.8, a probability level of 0.05, an effect size f of 0.26 with a main effect on REE, 32 young adult women were enrolled for this study. The present investigation was designed as a longitudinal controlled trial.

**Figure 1:**
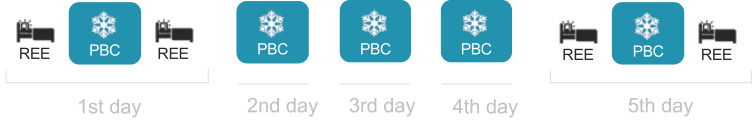
Flow-chart of the study. Obese and lean women underwent multiple exposures of Partial-Body Cryostimulation PBC (*n* = 5). Their Resting Energy Expenditure (REE) was indirectly derived prior and upon completion of the first and fifth day of PBC session.

Subjects underwent a morning single session of PBC/day for 5 consecutive days. On day 1 and 5, before and after each PBC, participants’ REE was assessed by indirect calorimetry. Study design is depicted in Figure 1.

Before the intervention, a physician conducted a physical examination of the subjects to exclude any contraindication to PBC. Pharmacological therapy was assessed before the study. People undergoing any pharmacological therapy on a regular basis or taking medication influencing metabolic homeostasis were excluded. In addition, people who had been exposed to cryostimulation procedures within the last 6 months, smokers, alcohol abusers, those following specific diets or taking supplements that may affect the metabolic rate, were also excluded from the trial.

As to the energy balance not deriving from REE, subjects were screened for physical activity levels (IPAQ questionnaire [17]) and caloric and dietary intake (EPIC questionnaire [18,19]). Throughout the intervention, participants were encouraged to maintain their physical and dietary habits.

Subjects were given full information about the scope and the methodology of the study, which was approved but Ethics Committee of the Università degli Studi di Milano (Approval n° 9/15) in accordance with the Declaration of Helsinki. Finally, subjects signed a written informed consent to participate.

Anthropometrics and REE measurements. Subjects’ body weight and height were measured to the nearest of 0.1 kg and 1 mm, respectively, by using a commercial scale/stadiometer device (Tanita BC 571, Tanita corporation, Tokyo, Japan). BMI was calculated for each individual as weight (kg)/height (m)^2^ (Table 1).

REE was assessed employing a validated [20] indirect calorimeter device (Q-NRG, Cosmed, Rome, Italy) prior and right after PBC application exclusively on day 1 and day 5, of the intervention. Subjects were instructed to observe a proper lifestyle and dietary procedures (fasting, avoid any sport activity and coffee consumption) 24 h prior to the measurement. Participants were also instructed to attain to specific instructions during the exam for a correct execution of the procedure. All participants were studied at the same hour of the day, two subjects per day, from 7:00 a.m. to 8:00 a.m., approximately. The room was kept in a range of thermoneutrality (around 20–22 °C) through the modulating temperature and humidity percentage by means of an air conditioning apparatus. Subjects remained dressed and lying supine on medical examination bed for 10 min, before positioning an isolating plexiglass canopy over their head. After another 10 min period for acclimatization to the device and for equilibration of experimental parameters, started a 20 min recording period where participants remained motionless for the entire length of the exam, without closing their eyes and cross their legs. Respiratory gaseous exchanges ($\dot{V}$CO_2_ and $\dot{V}$O_2_) were measured. REE was estimated via software calculation (OMNIA, Cosmed, Rome, Italy); respiratory quotient (RQ) was also estimated as the ratio $\dot{V}$CO_2_/$\dot{V}$O_2_, in order to obtain data on the variation of substrate utilization, following the PBC procedure.

### 2.2. Partial-Body Cryostimulation Procedure

Participants were exposed one by one to very-low temperatures in a cryo-cabin (Cryomed Pro, Criomed, Ltd., Kherson, Ukraine), an open tank equipped with a mobile lift which allows to adjust the height of each subject, so the user is exposed to the cold dry air up to the shoulders, with the neck and the head out of the cabin. Each session followed the standard indications [13]: 150 s at a temperature raging from −130 to −170 °C. According to the manufacturer’s recommendations, each subject wore shorts, a pair of gloves, woolen sock and wooden clogs to prevent the occurrence of frostbites. Participants were instructed by a qualified operator to walk on the same spot and turn around continuously for all the time of the treatment.

### 2.3. Statistical Analysis

All data are expressed as means ± standard deviation (SD) except where differently indicated. REE results were reported as means of absolute values as well as change in percentage to baseline. Normality of the data was assessed by Shapiro–Wilk test and skewness and kurtosis indexes were also ensured to be both within the conventional cut-off [21]. Baseline characteristics between groups were tested by a two-tailed independent Student’s *t*-test.

Mixed model analyses of variance were applied on changes in REE, respiratory quotient (RQ), $\dot{V}$CO_2_ and $\dot{V}$O_2_, to compare the effects of PBC procedure in the two groups (leans vs. obese), the two sessions (day 1 vs. day 5) and the time (pre-PBC vs. post-PBC). This approach was chosen to take into account the intrinsic (and uncontrolled) variability among the participants, which was considered everywhere as a random factor.

Post hoc pair-wise comparisons were conducted utilizing Newman–Keul test when interaction effects were demonstrated. The sphericity assumption was examined using Mauchly’s test.

Partial eta squared (η_p_^2^) effect sizes (ES) were determined and interpreted using the following cutoffs: small effect, η_p_^2^  ≤  0.03; medium/moderate effect, 0.03  < η_p_^2^  <  0.10; large effect, 0.10  ≤  η_p_^2^  <  0.20; very large effect, η_p_^2^  ≥  0.20 [22]. For all analyses, a *p* value less than 0.05 was considered statistically significant.

Analyses were carried out with the Statistical Package SPSS version 26 for Mac (Armonk, NY, USA; IBM Corp.), GraphPad Prism 8 (San Diego, CA, USA) and Excel version 16.32 for Mac (Microsoft, Redmond, WA, USA).

## 3. Results

Fifteen obese individuals (BMI 31 ± 1.6 kg/m^2^) and 16 lean controls (BMI 22 ± 1.7 kg/m^2^) were admitted to this study. One obese subject turned out to be outlier [23] and therefore was not included in the statistical analysis. Subjects’ anthropometrical and energy characteristics at baseline are summarized in Table 1.

Baseline REE resulted to be higher in the obese women with respect to lean controls (1610 ± 110 kcal vs 1538 ± 111 kcal; *p* < 0.0001). Both groups were sedentary according to IPAQ questionnaire. No significant difference was found as to the estimated dietary intake between groups.

### 3.1. Multiple PBC Induced Incremental Response in REE in Lean and Obese Women

We investigated whether sessions of PBC carried out on five consecutive days might produce greater increase in REE in lean and obese subjects.

REE significantly increased from day 1 to day 5 (session: F_1,29_ = 21.86; *p* = 0.00006, η_p_^2^ = 0.43) and from pre- to post-PBC (time: F_1,29_ = 5.23; *p* = 0.03, η_p_^2^ = 0.15). Furthermore, a significant session × time interaction was found (F_1,29_ = 5.58; *p* = 0.025, η_p_^2^ = 0.16), indicating that REE increased significantly following the first session of PBC (*p* = 0.0007). Changes of REE after the fifth session of PBC comparing to before fifth session were not statistically significant. However, REE values registered before and after the fifth session of PBC were significantly higher than those registered on session 1 (0.001 < *p* <0.05).

**Figure 2:**
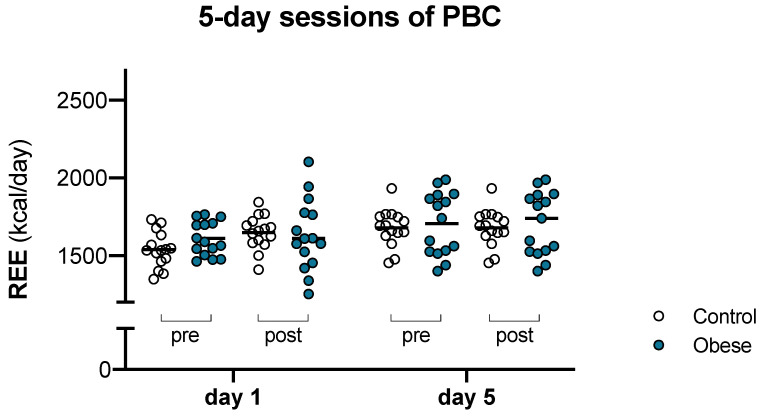
Effects of 5 days of cryostimulation sessions on the REE recorded before and after PBC, on day 1 and day 5, in obese and lean women (controls). Statistically significant comparisons are indicated in the results section.

In lean women, REE values registered before PBC augmented by 8.2% from day 1 to day 5 (1538 ± 111 vs 1665 ± 106 kcal/day; Figure 2). Likewise, in obese women, REE values registered before PBC augmented by 5.5% from day 1 to day 5 (1610 ± 110 vs 1698 ± 142 kcal/day; Figure 2).

Mean ± SD data are reported in Table 2 and in Supplementary File (Table S1).

### 3.2. PBC Induced a Shift in Substrate Oxidation

$\dot{V}$CO_2_ significantly increased from pre- to post-PBC (F_1,29_ = 51.26; *p* = 0.00001, η_p_^2^ = 0.64). Moreover, a significant interaction session×time×group was detected for $\dot{V}$CO_2_ (F_1,29_ = 7.948; *p* = 0.0085, η_p_^2^ = 0.22). In lean women, $\dot{V}$CO_2_ increased after the first session of PBC comparing to before the first session (*p* = 0.0001, Table 2). The same pattern occurred in session 5 (*p* = 0.006, Table 2). Further, $\dot{V}$CO_2_ registered before the first session of PBC was lower than that one registered before the fifth session of PBC (*p* = 0.017).

In the obese women, $\dot{V}$CO_2_ increased significantly after PBC in both sessions (day 1: *p* = 0.0009; Day 5: *p* = 0.0002. Table 2). Finally, $\dot{V}$CO_2_ registered after the first session of PBC was lower than that one registered after the fifth session of PBC (*p* = 0.01). 

$\dot{V}$O_2_ significantly increased from day 1 to day 5 (F_1,29_ = 26.92; *p* = 0.00002, η_p_^2^ = 0.48). Furthermore, a significant session×time interaction (F_1,29_ = 4.66; *p* = 0.039, η_p_^2^ = 0.14) showed that $\dot{V}$O_2_ augmented after the first session of PBC (*p* = 0.047, Table 2). Changes of $\dot{V}$O_2_ after the fifth session of PBC comparing to before fifth session were not statistically significant. However, $\dot{V}$O_2_ values registered before and after the fifth session of PBC were significantly higher than those registered on session 1 (always *p* < 0.001, Table 2).

RQ values significantly increased from pre- to post-PBC (F_1,29_ = 51.61; *p* < 0.000001, η_p_^2^ = 0.64).

Mean ± SD data are reported in Table 2 and in Supplementary File (Tables S2–S4).

## 4. Discussion

To our knowledge, this is the first research aimed at investigating the effect of PBC on REE in lean and obese subjects. Results achieved suggest that a single exposure to controlled cold temperature is sufficient to increase REE by a significant extent. Diversified cold applications elicit an incremental thermogenic effect. This is in accordance with interventions demonstrating adipose tissue loss upon repeated sessions of local cooling [24]. Our results can be seen as a step further in the comprehension of body response to PBC, as to substantiate its utilization in clinical practice.

Obese subjects at baseline presented higher REE values compared to lean subjects (respectively 1610 ± 110 vs 1538 ± 111 kcal/day), as evidenced elsewhere [25]. Both groups reported a dietary energy intake below the measured REE, which is a common finding, especially among obese individuals [26].

Cold-induced thermogenesis can be increased by leveraging on both ST and NST. Different tissues are able to increase their aerobic capacity in the effort to face temperature drop, contributing to increase NST [27]. However, BAT and muscle seem to account for a larger magnitude of cold-induced thermogenesis [10,28]. Shivering has not been observed in our intervention neither during nor before the procedure, which suggests that the effect recorded is virtually attributable to the activation of the NST; however, based on variables examined and data considered, the contribution of ST to REE increment cannot be completely ruled out.

Brown and brown-like adipocytes promote energy expenditure through sympathetic-activated thermogenesis which upregulates the uncoupled protein response [4]. Midterm cold exposure was demonstrated to induce a BAT-mediated oxygen consumption in short and long term (+0.1–0.6% and +0.5–2.3% of whole-body) [29]. Four-week cold exposure led to 45% increase of BAT volume and 182% of substrate oxidation from baseline [30]. Previous investigations have shown that cryostimulation procedure was found to increase irisin in lean and obese individuals [15,31]. Irisin is a recently discovered hormone, secreted from skeletal muscles in response to physical activity; it is thought to play a role in boosting thermogenesis in human, thus mediating part of the exercise-induced weight loss. Irisin is currently considered an indirect marker of browning since stimulates the activation of p38 and ERK signaling, enabling UCP-1 expression in adipocytes [32].

In our experiment we found that multiple PBC applications were able to increase REE to a significative extent in both lean and obese women. Nevertheless, obese people seemed to be less responsive compared to lean controls. Thereby, the higher adiposity in obese subjects seems to blunt the cold-induced increase in REE. In the absence of further substantiating data, we have advanced several possible explanations:

- Brown and Brown-Like Adipocytes Are Less Represented in Obese Compared to Lean Individuals

PET studies demonstrated that BAT activity is inversely related to BMI and lower in obese subjects than in lean subjects [33,34]. As mentioned, energy excess and overnutrition stimulate the brown-to-white conversion [4], reducing the overall mass of brown or brown-like cells suitable to cold-induced metabolic induction. This occurs in the interscapular area as well as single cells interspersed among white deposits [4] with the purpose of augmenting the storage of triglycerides. White adipocytes would then replace brown adipocytes in adipose tissue niches, thus diminishing de facto the quota of metabolically active adipocytes possible augmenting REE. In addition to volume reduction, a diminished activity and biogenesis of mitochondrial BAT in obese can account for the lower response to cold of these individuals [10,35].

- *Fat-Free Mass (FFM) and Chiefly Skeletal Muscles Are Likely to Provide a Great Contribution to Cold-Induced Thermogenesis*

Beside BAT, an increase in substrate oxidation has been demonstrated in different tissues, upon cold exposure. Recent advances suggest that FFM is pivotal for cold-induced NST [10,27]. In skeletal muscle, several mechanisms are activated during cold exposure to generate futile cycles including proton leak, substrates synthesis and degradation and sarcoplasmic reticulum Ca^2+^ ATPase (SERCA), with the result of augmenting heat production and energy expenditure [10]. Proton leak accounts for approximately 10% of REE in normal conditions and is generated through UCP-3 in muscle [10]. Recent data show that sarcolipin/SERCA interaction plays an important role in muscle-induced thermogenesis and REE increase [28,36]. Molecules such as sarcolipin are demonstrated to trigger SERCA uncoupling activity, increasing ATP hydrolysis and heat production during cold exposure.

- *Dysfunctional WAT Impairs Muscle Metabolism Likely Altering Obese Subjects’ Thermogenetic Capacity*

Obese animals display a reduced thermogenetic capacity in response to cooling [37]. Similarly, in a study of Jung et al., obese subjects with a family history of obesity showed a reduced metabolic response to noradrenaline administration compared with lean subjects [35]. In obesity, the pathological accumulation of lipids in skeletal muscle and the low-grade inflammation impairs insulin signaling [38] and tissue proper glucose uptake and utilization. Similarly, skeletal muscle lipid oxidation is impaired in obesity [39]. Obesity-related mitochondrial dysfunction could be also implicated in the lower rate of substrate oxidation (as testified by the lower increase in $\dot{V}$O_2_ in the obese group) [40]. Furthermore, the activity of SERCA was shown to be impaired in animal models of obesity and metabolic syndrome [41,42], potentially representing an adjunctive factor accounting for the poorer response to PBC-induced thermogenesis in the group of obese-women. Thereby, dysmetabolism in obesity could possibly affect muscle substrate oxidation [43], de facto preventing a prompt thermogenic response to cold exposure.

- *WAT Response to Cooling Is Probably Poorly Involved in REE Increase in Short-Term Exposure*

Energy expenditure following cold exposure can also occur in WAT independently of BAT activation [34]. Both WAT and BAT are capable of metabolizing energy by pathways independent of UCP1 [44]. The activation of resident immune cells upon cold exposure is also suggested to enhance WAT thermogenesis [10]. Moreover, WAT was demonstrated to increase energy expenditure and mitochondrial activity in response to cold [45]. Nonetheless, in our study, WAT contribution seems to be limited. Since no insulating effect of adipose tissue has been evidenced in obesity [46], the dysfunctional metabolic phenotype and the scarcity of brown-like adipocytes in WAT of obese are likely to account for its poorly responsiveness to short-term cooling exposure.

Given a higher increase of REE of lean women with respect to obese ones, when comparing REE_pre-PBC on day 5 vs day 1, it is possible that the relatively higher FFM/FM in lean subjects as well as the impairment of WAT, BAT and muscle metabolism could be responsible for the putative metabolic refractoriness of obese subjects. At this stage, the lack of detailed subjects’ body composition impedes to draw that line of reasoning. Otherwise, one may speculate that repeated sessions of PBC tends to engage BAT to a larger extent, thus promoting a shifting from muscle to BAT-induced thermogenesis.

RQ increment following each PBC session, suggests an augmented carbohydrate (CHO) utilization. RQ through a quantitative assessment of respiratory gases ($\dot{V}$CO_2_/$\dot{V}$O_2_), provides a qualitative, indirect estimation of whole-body substrate oxidation, indicating a prevalent CHO utilization (when the ratio is close to 1.00) or a larger percentage of lipid oxidation (when the ratio is close to 0.7). Since food habits did not change during intervention, RQ variation is attributable to cold-induced substrate shifting. The shifting to CHO oxidation is consistent with previous studies on controlled cold exposure [15,31] and investigations showing a scarce lipid mobilization upon local cooling [24]. BAT contains a significant quota of endogenous glycogen [4] and cold exposure in murine model was demonstrated to significantly increase BAT glucose uptake [47]. BAT of triglyceride-lacking mice was shown to employ circulating glucose and stored glycogen to fuel thermogenesis in place of lipids [48,49]. Furthermore, cold-triggered skeletal muscle activation is likely to contribute to glucose oxidation [10]. Conversely, in longer exposures CHO utilization can be replaced by lipid oxidation as suggested by Blondin et al. [30]. These findings then indicate that probably cold exposure exerts only a modest effect on triglyceride cleavage and mobilization in WAT, as suggested by other authors [24]. The higher RQ shifting in obese women (lean and obese women presented the same baseline RQ value) suggests a preferential relative CHO oxidation in obesity. This finding is supported by the occurrence of impaired fat oxidation in skeletal muscle [39] in obesity and the scarce lipid mobilization observed in obese subjects undergoing local cooling [24]. The impaired fat oxidation can therefore fuel glycogen and/or glucose utilization in BAT and muscles of obese individuals. Our RQ data also suggest that the impaired lipid oxidation in obese are not efficaciously corrected by short-term PBC exposure. However, a relative increase in lipids and protein oxidation cannot be completely ruled out based on RQ data recorded.

The present work was intended as an explorative study to assess the variation of REE upon PBC application. As such, some limitations can be evidenced. First of all, part of the intervention was restricted to only women, therefore lacking generalizability. Women present higher WAT and BAT volumes and a smaller ratio visceral/subcutaneous WAT, with a different pattern of regional distribution compared to men [33,50]. The lack of data on BAT and WAT volumes, adipose tissue specific cytokines and markers of browning (e.g., irisin) further precluded the possibility of an in-depth discussion on BAT and WAT response to PBC as well as speculating on the possible causes of REE increase. Finally, as suggested by previous investigations [15,24], a longer period of PBC application might have likely resulted in a more effective induction of REE. Loap et al. [24], for example, observed that weight loss continued to occur in obese people over a period of 3 months after discontinuing cold exposure. In this view, further investigations are encouraged to test the effect of longer exposure periods.

No adverse events were reported in this study. Future studies are advised to focus on the possible side effects elicited by repeated PBC treatments, evidencing the time-point in which beneficial effects are overruled by possible (if any) side effects.

## 5. Conclusions

To our knowledge, this study was the first to demonstrate that either single or multiple PBC sessions significantly induce the augment of REE in lean and obese women, albeit demonstrating differential effectiveness. Additional studies are granted to investigate the metabolic results secondary to longer treatment periods, mainly focusing on the time-duration of the REE increase gained after PBC. Since evidence on the topic is still embryonic, further evaluations regarding repeated sessions of PBC therapy in obese subjects are advisable in order to substantiate the employment of this procedure as a possible strategy to significatively augment REE, in this class of patients.

## Figures and Tables

**Table 1:** Characteristics of the study subjects.

	Lean *(n = 16)*	Obese *(n = 15)*	*p* Value
Age (years)	40.4 ± 6	42.7 ± 10.4	0.46
Height (cm)	162.6 ± 5.5	163.7 ± 6.8	0.64
Weight (kg)	58.3 ± 4.5	83.2 ± 7.3	<0.0001
BMI (kg/m^2^)	22 ± 1.7	31 ± 1.6	<0.0001
Basal REE * (kcal/day)	1538 ± 111	1610 ± 110	<0.0001
Physical Activity (MET **-min/week)	1760 ± 1660	2149 ± 934	0.7
Dietary intake (kcal/day)	1254 ± 191	1432 ± 820	0.67

Data are shown as mean ± SD. * Resting Energy Expenditure. ** Metabolic Equivalent of Task.

**Table 2:** Study-subjects’ results throughout the entire experimental timespan.

	Lean				Obese			
	Day 1		Day 5		Day 1		Day 5	
	Pre	Post	Pre	Post	Pre	Post	Pre	Post
$\dot{V}$O_2_ (mL/min)	224 ± 15	235 ± 17	244 ± 15	242 ± 17	234 ± 17	234 ± 37	248 ± 21	244 ± 31
$\dot{V}$CO_2_ (mL/min)	176 ± 20	203 ± 16	186 ± 20	197 ± 20	186 ± 17	201 ± 23	193 ± 23	212 ± 24
RQ ($\dot{V}$CO_2_/$\dot{V}$O_2_)	0.79 ± 0.06	0.86 ± 0.08	0.76 ± 0.07	0.82 ± 0.07	0.80 ± 0.08	0.87 ± 0.11	0.78 ± 0.09	0.87 ± 0.07
REE (kcal/day)	1538 ± 111	1645 ± 107	1665 ± 106	1676 ± 115	1610 ± 110	1632 ± 229	1698 ± 142	1706 ± 203

Data are shown as mean ± SD.

## Data Availability

The data presented in this study are available on request from the corresponding author.

## Supplementary Materials

The following are available online at https://www.mdpi.com/article/10.3390/ijerph18084127/s1, Table S1: mx linear_mix_mod on resting energy expenditure (REE); Table S2: mx linear_mix_mod on respirartory quotient (RQ); Table S3. mx linear_mix_mod on $\dot{V}$O_2_; Table S4. mx linear_mix_mod on $\dot{V}$CO_2_.
