# Supplementary material for: Partial-Body Cryostimulation Increases Resting Energy Expenditure in Lean and Obese Women
Source: Int J Environ Res Public Health. 2021 Apr 14;18(8):4127. doi: 10.3390/ijerph18084127 (PMC8070728; doi:10.3390/ijerph18084127)
Supplement: Supplementary file 1 [file ijerph-18-04127-s001.pdf]

Table S1. mx linear_mix_mod on resting energy expenditure (REE).

	gl effect	MS effect	gl error	MS error	F	p	partial eta squared
group	1	29190.7	29.00000	63941.83	0.45652	0.505	0.015
session	1	198000.2	29.00000	9057.42	21.86055	0.00006	0.430
time	1	43668.8	29.00000	8350.76	5.22932	0.030	0.153
group*session	1	20.9	29.00000	9057.42	0.00230	0.962	0.000
group*time	1	15062.3	29.00000	8350.76	1.80370	0.190	0.059
session*time	1	25236.8	29.00000	4522.86	5.57983	0.025	0.161
group*session*time	1	13101.6	29.00000	4522.86	2.89676	0.099	0.091

					mean	st dev	st err	lmt cnf - 0.95%	lmt cnf + 0.95%
norm	1	pre		16	1538	111	28	1478.457	1596.668
norm	1	post		16	1645	107	27	1587.707	1702.043
norm	5	pre		16	1665	106	27	1608.394	1721.856
norm	5	post		16	1676	115	29	1614.055	1737.070
ob	1	pre		15	1610	110	28	1549.256	1670.877
ob	1	post		15	1632	229	59	1505.121	1759.146
ob	5	pre		15	1698	142	37	1619.484	1776.783
ob	5	post		15	1706	203	52	1593.314	1817.886

Table S2. mx linear_mix_mod on respirartory quotient (RQ).

	gl effect	MS effect	gl error	MS error	F	p	partial eta squared
group	1	0.016546	29.00000	0.015161	1.09135	0.305	0.036
session	1	0.016261	29.00000	0.004833	3.36430	0.077	0.104
time	1	0.172132	29.00000	0.003335	51.60803	0.00000	0.640
group*session	1	0.005618	29.00000	0.004833	1.16226	0.290	0.039
group*time	1	0.002092	29.00000	0.003335	0.62716	0.435	0.021
session*time	1	0.000261	29.00000	0.001612	0.16211	0.690	0.006
group*session*time	1	0.003247	29.00000	0.001612	2.01450	0.166	0.065

					mean	st dev	st err	lmt cnf - 0.95%	lmt cnf + 0.95%
norm	1	pre		16	0.79	0.06	0.02	0.75	0.82
norm	1	post		16	0.86	0.08	0.02	0.82	0.90
norm	5	pre		16	0.76	0.07	0.02	0.73	0.80
norm	5	post		16	0.82	0.07	0.02	0.78	0.85
ob	1	pre		15	0.80	0.08	0.02	0.75	0.84
ob	1	post		15	0.87	0.11	0.03	0.81	0.93
ob	5	pre		15	0.78	0.09	0.02	0.73	0.83
ob	5	post		15	0.87	0.07	0.02	0.83	0.91

Table S3. mx linear_mix_mod on $\dot{V}O_2$.

	gl effect	MS effect	gl error	MS error	F	p	partial eta squared
group	1	379.129	29.00000	1513.189	0.25055	0.620	0.009
session	1	5033.065	29.00000	186.964	26.91991	0.000015	0.481
time	1	68.258	29.00000	193.920	0.35199	0.558	0.012
group*session	1	17.468	29.00000	186.964	0.09343	0.762	0.003
group*time	1	401.574	29.00000	193.920	2.07083	0.161	0.067
session*time	1	528.516	29.00000	113.328	4.66360	0.039	0.139
group*session*time	1	171.474	29.00000	113.328	1.51308	0.229	0.050

					media	dev st	err st	lmt cnf - 0.95%	lmt cnf + 0.95%
norm	1	pre		16	224	15	4	215.91	232.22
norm	1	post		16	235	17	4	226.40	244.47
norm	5	pre		16	244	15	4	235.86	252.02
norm	5	post		16	243	17	4	233.39	251.61
ob	1	pre		15	234	17	4	224.63	243.91
ob	1	post		15	234	37	9	213.50	253.97
ob	5	pre		15	248	21	6	236.04	259.83
ob	5	post		15	244	31	8	227.02	260.98

Table S4. mx linear_mix_mod on $\dot{V}CO_2$.

	gl effect	MS effect	gl error	MS error	F	p	partial eta squared
group	1	1596.09	29.00000	1051.765	1.51754	0.228	0.050
session	1	857.06	29.00000	357.443	2.39776	0.132	0.076
time	1	10080.03	29.00000	196.638	51.26189	0.000	0.639
group*session	1	324.59	29.00000	357.443	0.90808	0.349	0.030
group*time	1	30.97	29.00000	196.638	0.15749	0.694	0.005
session*time	1	309.81	29.00000	87.601	3.53656	0.070	0.109
group*session*time	1	696.26	29.00000	87.601	7.94807	0.009	0.215

					media	dev st	err st	lmt cnf - 0.95%	lmt cnf + 0.95%
norm	1	pre		16	176	20	5	165.64	186.86
norm	1	post		16	203	16	4	194.51	211.49
norm	5	pre		16	186	20	5	175.49	196.76
norm	5	post		16	197	20	5	186.89	207.86
ob	1	pre		15	186	17	4	176.34	195.53
ob	1	post		15	201	23	6	188.43	213.97
ob	5	pre		15	193	23	6	179.94	205.66
ob	5	post		15	212	24	6	197.98	225.09
